# On-Machine Precision Truing and Error Compensation of Cup-Shaped Diamond Grinding Wheels with Arc-Shaped Cutting Edge

**DOI:** 10.3390/mi16091050

**Published:** 2025-09-15

**Authors:** Yawen Guo, Ziqiang Yin

**Affiliations:** Guangdong Provincial Key Laboratory for Micro-Nano Manufacturing Technology and Equipment, School of Electro-Mechanical Engineering, Guangdong University of Technology, Guangzhou 510000, China; 1112101013@mail2.gdut.edu.cn

**Keywords:** cup-shaped grinding wheels with arc-shaped edges, on-machine truing, error compensation, ultra-precision grinding

## Abstract

The cup-shaped grinding wheels with arc-shaped edges provide a satisfactory precision grinding solution for high-accuracy optical surfaces on hard and brittle materials. However, the complex profile of the arc-shaped edges of cup-shaped grinding wheels makes them challenging to truing. This paper proposes an on-machine truing technique targeting cup-shaped grinding wheels with arc-shaped cutting edge. First, a mathematical model was established to simulate the three-axis of on-machine truing the arc-shaped cutting edge using a diamond roller. Based on this model, a theoretical analysis is conducted to investigate the impact of tool setting errors, measurement errors of the diamond roller, and the pose error on truing accuracy. A compensation method was proposed, and experimental results validated its effectiveness. To investigate the grinding performance of cup-shaped grinding wheels after truing, a complex component is ground using a truing diamond grinding wheel. The experimental results demonstrate that this method enables precise on-machine truing of the arc-shaped edges of cup-shaped grinding wheels and is efficient. The average dimensional accuracy of the grinding wheel’s arc-shaped edge is reduced to 1.5 μm, with the profile accuracy (PV) of 0.89 μm.

## 1. Introduction

As the industry develops, the demand for functional surfaces continues to increase, particularly in optical applications, where hard and brittle material surfaces need to meet requirements for high-profile accuracy [1,2,3]. Cup-shaped diamond grinding wheels can be used for aspherical and free-form surfaces, playing a vital role in the optical manufacturing industry [4,5]. The shape of the cutting edge of cup-shaped grinding wheels significantly affects their grinding performance and the suitability of their applications. When the shape of the cutting edge is straight or inclined, the grinding force can be distributed evenly, thereby reducing vibration caused by the grinding process. However, this is only suitable for grinding workpieces with a simple profile. Cup-shaped grinding wheels with arc-shaped cutting edges have strong adaptability in workpiece dimensions, distribute cutting forces evenly, have a long service life, and exhibit high adaptability to workpiece profiles. They also have significant advantages in grinding complex structures such as aspherical and free-form surfaces. Therefore, cup-shaped grinding wheels with arc-shaped cutting edges are widely used to grind optical components. Resin-bonded diamond grinding wheels are effective for achieving good surface quality during grinding, but they are more prone to wear [6]. High-precision workpiece profiles can only be achieved by periodically truing the grinding edges of the diamond grinding wheel throughout the grinding process [7,8]. Moreover, the truing process for cup-shaped grinding wheels with arc-shaped cutting edges is complex. Due to the straight-edged and inclined-edged cup wheels employing single-point grinding during the machining process, leading to accelerated edge wear. Conversely, cup-shaped grinding wheels with arc-shaped edges engage more grinding areas during complex surface machining, making them less susceptible to dimensional error caused by wheel wear. However, any profile errors in the wheel are transferred to the workpiece profile. Consequently, cup-shaped grinding wheels with arc-shaped edges require higher precision after truing to minimise the impact of wheel profile errors on the workpiece. Therefore, the on-machine truing and error compensation methods for cup-shaped grinding wheels with arc-shaped edges are essential for achieving ultra-precision grinding [9]. The truing of grinding wheels aims to eliminate errors caused by factors such as installation, manufacturing, and grinding wheel wear [10]. This is crucial for achieving good surface accuracy and quality [11,12]. There are four common methods of truing resin-bonded diamond grinding wheels [13]. Single-point diamond pen truing is a simple method for truing the profile of grinding wheels [14], but it suffers from rapid wear of the diamond pen when truing large grinding wheels. Additionally, single-point diamond pens are not suitable for truing grinding wheels with complex profiles [15]. Pulse laser can truing grinding wheels in two directions [16,17]. Although it can be used for truing resin-bonded grinding wheels, the accuracy of the final grinding wheel shape is typically unstable and unreliable. While abrasive block truing makes it difficult to control the profile accuracy of the grinding wheel after truing, this is due to the abrasive block being prone to wear [18,19]. Diamond rollers can achieve grinding wheel truing through the combined effect of grinding and rolling at high speeds [20]. Diamond roller truing generally uses coarse-grained electroplated diamond grinding wheels, which make the rollers wear-resistant, and it is easy to control the shape of the grinding wheel edge, resulting in high truing efficiency [21]. This makes it the preferred method for high-precision truing of cup-shaped grinding wheels with arc-shaped cutting edges.

The truing method of cup-shaped diamond grinding wheels with arc-shaped cutting edges is exceedingly rare to report. Chen et al. [22] proposed a method for grinding wheels using an on-machine truing resin and metal-bonded rotating green silicon carbide rod (ORGCR), employing the wheel’s interpolation motion to form circular arcs of arbitrary radii. Experimental verification demonstrated that this approach can enhance the contour accuracy of pre-dressed grinding wheels by approximately 90%. Wang et al. [23] employed an electrical discharge truing method to control errors within 2 µm for metal-bonded spherical grinding wheels, though this requires matching optimal process parameters. Zhu et al. [24] developed a spiral interpolation forming method for truing curved diamond grinding wheels. The process enables controlled wear of the forming wheel during truing, achieving a profile accuracy of 5 µm (PV) for the grinding wheel. Qiao et al. [25] proposed a novel dressing/sharpening method employing a cup-shaped GC grinding wheel as the dressing tool and applying axial ultrasonic vibration to diamond grinding wheels. This method improved the shape accuracy, abrasive sharpness, and effective quantity of the dressed grinding wheels. Lu et al. [26] proposed dry electrical discharge truing (DEDT) technology for dressing metal-bonded diamond grinding wheels. By optimising electrical discharge dressing parameters, the PV value and RMS value of the V-shaped tip could be reduced to 23.2 µm and 5.06 µm, respectively. Meng et al. [27] employed a novel laser wheel dressing method incorporating a spray-mist assistant (SMA) for the dressing of electroplated diamond grinding wheels. This approach resolved the graphitisation of diamonds following laser dressing. Guo et al. [28] proposed a pulsed laser method for dressing V-shaped coarse-grained electroplated cubic boron nitride (CBN) grinding wheels. By analysing the effects of dressing depth, wheel rotational speed, laser power, and dressing time, the V-shaped wheel angle achieved 90.15° (deviation 0.02°), with a bottom radius of 53 µm (less than 100 µm). Laser dressing yielded significantly lower surface roughness and grinding force than mechanical dressing, while reducing dressing time by 72% to 83%. Wang et al. [29] conducted research into the wear mechanism of electroplated diamond abrasives for precision dressing on curved diamond grinding wheels, proposing that the optimal grain size of electroplated diamond grinding wheels is pivotal to enhancing the contour accuracy of curved diamond grinding wheel correction. Employing a dressing wheel with D213 μm grain size, the radial runout error and cross-sectional arc contour error of a D35 μm mixed-bonded curved diamond grinding wheel were successfully reduced to 1.9 μm and 10 μm, respectively. Most of the above studies have focused on investigating the effects of truing methods and machining parameters on results, with few exploring the relationship between errors during machining and profile accuracy. Therefore, researching precision truing methods for the cup-shaped diamond grinding wheels with the arc-shaped cutting edges is of significant importance for further improving the forming accuracy of workpiece surfaces and reducing the machining allowance in subsequent polishing processes.

This paper proposes a novel on-machine truing method to address the significant challenges in truing the cup-shaped diamond grinding wheel with arc-shaped cutting edges. By establishing a profile error model, the study focuses on analysing the impact of pose errors and tool setting errors during machining on the profile accuracy of the grinding wheel. Based on the above model analysis, corresponding compensation methods are proposed. Experimental results validate the effectiveness of this truing method.

## 2. Truing Principle of Arc-Shaped Cutting Edge of Grinding Wheels

**Figure 1:**
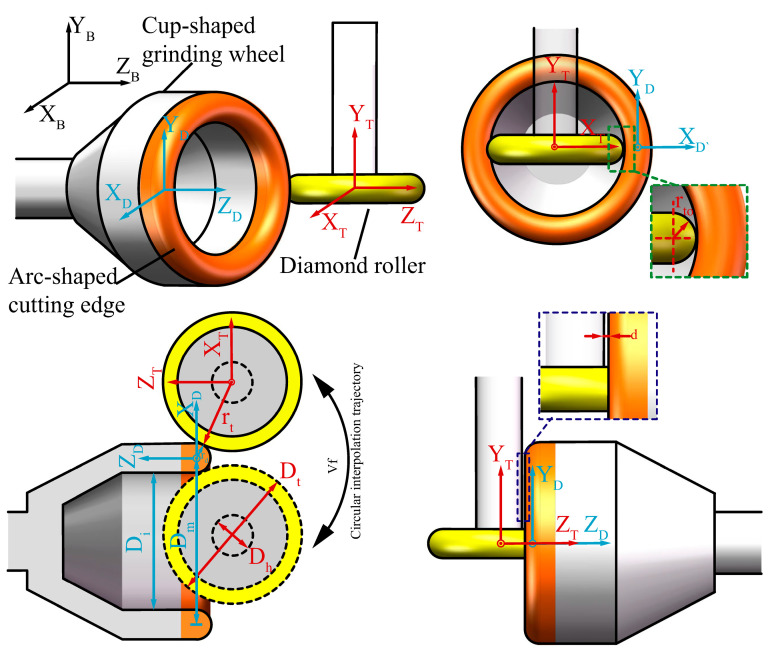
Truing principle of a diamond grinding wheel.

Figure 1 shows the principle of truing the arc-shaped cutting edge of the cup-shaped diamond grinding wheel. The diamond roller is driven by an electric spindle, which is fixed and rotates at a speed of ωd around the axis parallel to the Y-axis. The diamond grinding wheel is driven by a high-speed aerostatic bearing spindle, rotating at a speed of ωt and parallel to the Z-axis. The rotational axes of the grinding wheel spindle and the truing spindle are perpendicular to each other. The machining model incorporates two sets of coordinate systems: the diamond roller coordinate system (OX_T_Y_T_Z_T_), and the grinding wheel coordinate system (OX_D_Y_D_Z_D_).

During the truing process, the cup-shaped grinding wheel performs an arc interpolation motion around the diamond roller in the XZ plane. The cup-shaped grinding wheel cuts along the X-axis and Z-axis to locate the diamond roller’s border, determining the Z-axis’s starting position. Using this point as the reference point for interpolating the trajectory, after enveloping the complete machining path and shifting it by ΔV along the Z-axis, the preceding motion path is repeated until the desired grinding wheel contour is achieved.

**Figure 2:**
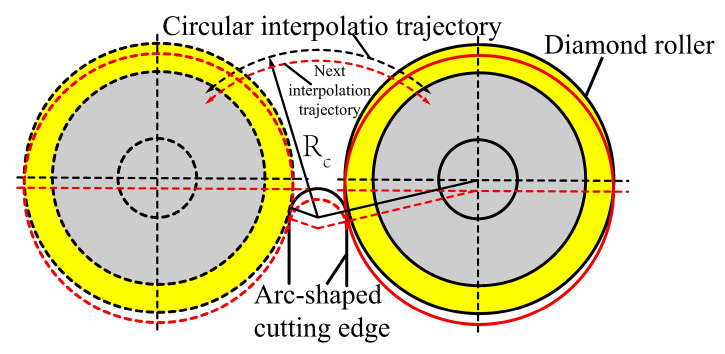
Geometric relationship between the cup-shaped grinding wheel and the diamond roller.

Figure 2 illustrates the geometric relationship between the arc-shaped cutting edge of the grinding wheel and the horizontal cross-section of the diamond roller during the truing process. R_c_ is the radius value of the grinding wheel truing trajectory, equal to the sum of the ideal radius of the grinding wheel with arc-shaped cutting edge r_d,_ and the radius value of the diamond roller r_t_. Changing the interpolation arc radius Rc can obtain any radius of the grinding wheel arc-shaped profile.

It is important to note that when using a diamond roller to truing a cup-shaped grinding wheel on-machine, to avoid interference between the cup-shaped grinding wheel and the diamond roller’s tool holder, and to complete the required truing range of the grinding wheel’s arc cutting edge, the geometric parameters D_t_ of the diamond roller are strictly constrained. When designing a cup-shaped grinding wheel truing system, the constraints on the diamond roller dimensions D_t_ and the cup-shaped grinding wheel’s arc edge dimensions can be described by Equation (1).

(1) $$2(\frac{(r_{d}-r_{d}\cdot \cos(180-\theta ))+\frac{D_{h}}{2}}{\sin(180-\theta )})<D_{t}<D_{i}$$

where r_d_ is the radius of the arc-shaped cutting edge of the grinding wheel; D_h_ is the diameter of the grinding wheel holder; D_t_ is the maximum diameter of the grinding wheel; D_m_ is the average diameter of the cup grinding wheel; D_i_ is the inner diameter of the cup-shaped grinding wheel; θ corresponds to the central angle of the arc-shaped truing range.

## 3. Analysis of Error Sources

Based on the principle of truing cup-shaped grinding wheels with diamond rollers, the main factors affecting the profile accuracy of the arc-shaped cutting edge include the pose error of the truing spindle and grinding wheel spindle, tool setting error, and dimensional and profile errors of the roller. During the truing process of cup-shaped grinding wheels, the diamond roller rotates at high speed, and the profile equivalent to the roller’s participation is an ideal arc. Theoretically, the arc-shaped cutting edge surface generated along the truing trajectory has no profile errors. However, the dimensional measurement errors of the diamond roller only affect the radius dimensions of the arc-shaped cutting edge, while the profile errors and dimensional errors of the diamond roller do not affect the profile accuracy of the truing grinding wheel. Tool setting error sources include the X, Y, and Z directions. In the Y-direction, it is sufficient for the horizontal generatrix of the cup-shaped grinding wheel’s arc-shaped cutting edge to coincide with the highest point of the roller in the Z-direction. The tool setting positions in the X and Z directions determine the starting position of the dressing process. The following sections discuss how truing spindle and grinding wheel spindle pose errors and tool setting errors affect the accuracy of truing cup-shaped grinding wheels.

### 3.1. Pose Error Between the Axis of the Truing Spindle and the Grinding Wheel Spindle

**Figure 3:**
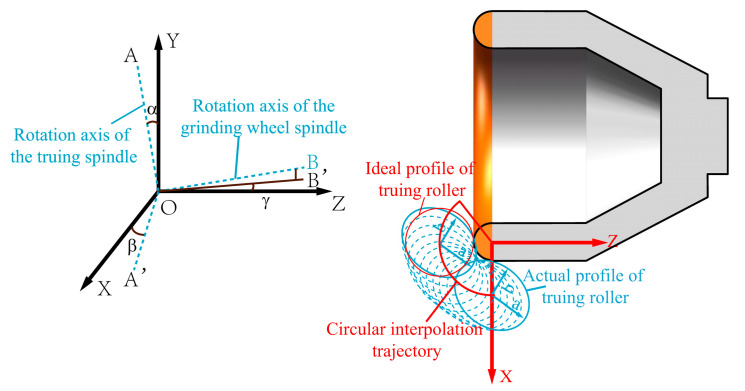
Truing spindle pose errors and their impact on the grinding wheel profile.

The pose errors are primarily determined by the installation accuracy of the truing spindle and the grinding wheel spindle. The pose error of the grinding wheel spindle is mainly the parallelism error between the projection of its rotational axis in the XOZ coordinate plane and the Z-direction. The pose error of the truing spindle is also the spatial relative position error between the rotational axis of the diamond roller and the cup-shaped grinding wheel. As shown in Figure 3, the coordinate system O-XYZ represents the machine tool coordinate system. All error analyses regarding the truing of diamond rollers are based on the O-XYZ coordinate system. Under ideal conditions, the rotational axis of the grinding wheel spindle coincides with the machine tool’s XOZ plane. It aligns along the Z-axis direction, while the rotational axis OA of the truing spindle is perpendicular to the axis of the grinding wheel spindle and aligned along the Y-axis direction. In actual conditions, the projection of the grinding wheel spindle rotation axis in the XOZ plane has an angular error γ relative to the Z direction. The angle error α of the truing axis deviating from the ideal position perpendicular to the grinding wheel spindle is such that the direction forms an angle β with the X-axis. OA’ is the projection of OA in the XOZ coordinate plane, and OB’ is the projection of OB in the XOZ coordinate plane. In this coordinate system, the ideal radius of the arc-shaped cutting edge of the cup-shaped grinding wheel is r_d_, with its centre coinciding with the origin O of the coordinate system. The radius of the roller is denoted as r_t_, and the radius of the truing motion programming trajectory is r_d_ + r_t_. The inclination of the grinding wheel causes the actual rolling contour of the roller to be an elliptical contour, with the central axis a and minor axis b of the ellipse expressed as in Equation (2).

(2) $$\left\{\begin{array}{c} a=\frac{r_{t}}{\cos(\alpha )}\\ b=r_{t} \end{array}\right.$$

At any point on the diamond roller centre arc interpolation trajectory, the ellipse can be described in Equation (3):

(3) $$\left\{\begin{array}{c} x=\frac{r_{t}}{\cos(\alpha )}\cdot \cos\beta \cdot \cos\theta -r_{d}\cdot \sin\beta \cdot \sin\theta -m\\ y=\frac{r_{t}}{\cos(\alpha )}\cdot \sin\beta \cdot \cos\theta -r_{d}\cdot \cos\beta \cdot \sin\theta -n \end{array}\right.$$

where

$$n=\sqrt{{\left(r_{d}+r_{t}\right)}^{2}-m^{2}}$$

θ represents the truing motion range, which is determined by the dimensions of the grinding wheel’s arc-shaped cutting edge and the diamond roller; m denotes the coordinate position of the roller’s centre on the X-axis at any point along the truing motion trajectory. Due to the pose error, the actual profile of the grinding wheel participating in truing is elliptical, with its dimensions and direction determined by the pose error angles α and β. Under the above error conditions, the arc-shaped edge of the cup-shaped grinding wheel is formed, resulting in an elliptical profile of the grinding wheel as it moves along the truing motion trajectory to obtain the inner envelope profile. Any point on this envelope curve passes through a specific elliptical position and is also the tangent point between the envelope curve and the elliptical curve at that position. The partial differential equations can be constructed as shown in Equation (4):

(4) $$\frac{\partial x}{\partial \theta }\cdot \frac{\partial y}{\partial m}-\frac{\partial x}{\partial m}\cdot \frac{\partial y}{\partial \theta }=0$$

The calculation shows that m is

(5) $$m={\left\{{\left[{\left(\frac{\cos\alpha \cdot \cos\beta \cdot \cos\theta -\sin\beta \cdot \sin\theta }{\cos\alpha \cdot \sin\beta \cdot \cos\theta +\cos\beta \cdot \sin\theta }\right)}^{-2}+1\right]}^{-1}\cdot {R_{C}}^{2}\right\}}^{\frac{1}{2}}$$

where

$$R_{c}=r_{d}+r_{t}$$

Substituting m into Equation (3) yields the equation for the contour line of the actual truing grinding wheel. r_td_ denotes the distance from any point on the actual truing grinding wheel profile to the theoretical centre point O of the cup-shaped grinding wheel arc edge. The difference between the maximum and minimum values of r_td_ is the contour error PV of the truing grinding wheel.

(6) $$\mathrm{PV}=\max(r_{\mathrm{td}})-\min(r_{\mathrm{td}})$$

Max (r_td_) and min (r_td_) represent the maximum and minimum values of r_td_.

**Figure 4:**
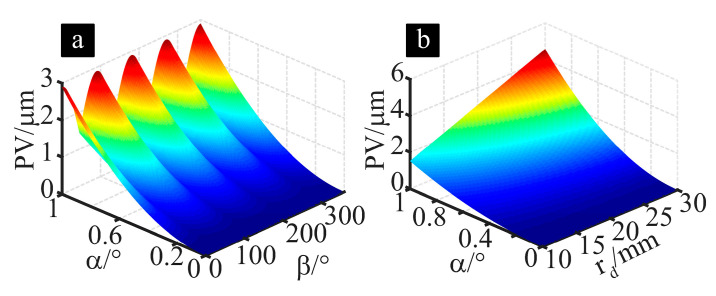
The effect of truing spindle pose error and the diamond roller’s dimension on the grinding wheels’ profile accuracy. (**a**) Effect of pose error of truing spindle. (**b**) Effect of diamond roller size.

Equations (3), (5) and (6) can be used to calculate the relationship between the arc-shaped cutting edge profile error PV of the cup-shaped grinding wheel and the truing axis pose errors α and β, as well as their sensitivity in different directions. Figure 4a shows the influence of truing spindle position errors α and β on the profile error of the arc-shaped grinding wheel edge. Clearly, as the angle α between the truing spindle rotation axis and the ideal position increases, the profile error of the cup-shaped grinding wheel’s arc-shaped edge increases. However, when the tilt direction of the positional error is parallel or perpendicular to the axis of the grinding wheel, the positional error has the greatest impact on the surface profile error of the arc-shaped cutting edge. Furthermore, calculations indicate that selecting a larger-diameter diamond roller amplifies the influence of truing spindle pose errors on the profile error of the grinding wheel’s arc-shaped edge, as shown in Figure 4b. Therefore, when using cup-shaped grinding wheels for grinding, higher machining accuracy can be achieved by adjusting and limiting the pose error of the truing spindle and the dimensions of the diamond roller according to the profile error requirements of the workpiece. For example, when the diamond roller radius is selected as 19 mm, if the profile error of the grinding wheel introduced by the truing spindle pose error is required to be less than 1 μm, then the installation error angle α of the truing spindle in the direction parallel or perpendicular to the grinding wheel spindle rotation axis should be less than 0.59°.

**Figure 5:**
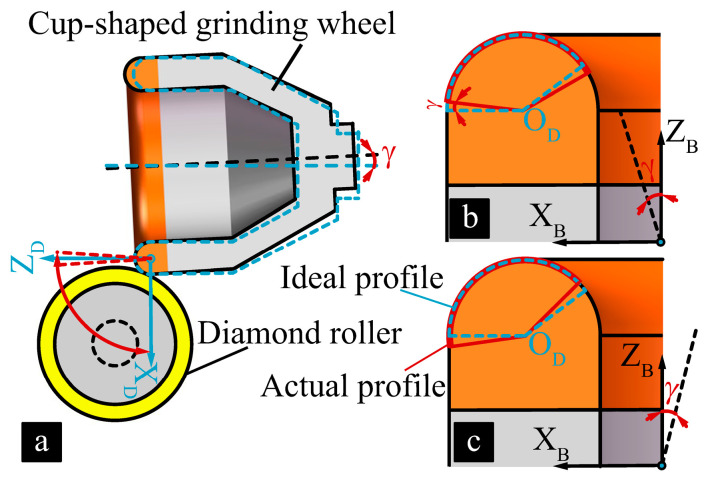
The effect of the grinding wheel spindle swing angle error γ on the profile error. (**a**) Schematic diagram of grinding wheel swing angle error. (**b**) γ < 0. (**c**) γ > 0.

The effect of the grinding wheel spindle swing angle error γ on the profile error of the cup-shaped grinding wheel arc cutting edge is shown in Figure 5. When the pose error γ of the grinding wheel spindle deviates towards the opposite side of the truing trajectory, the initial position of the actual grinding wheel profile is higher than the centre of the ideal profile. Referring to Figure 5a, it can be seen that the centre angle of the grinding wheel’s arc cutting edge is still θ, and the enveloping machined surface is an ideal arc surface. However, overcutting may occur at the end of the truing trajectory. When the grinding wheel spindle position error γ deviates toward the same side of the truing trajectory, the actual grinding wheel profile has an initial position lower than the centre of the ideal profile. The centre angle of the arc cutting edge is θ, as shown in Figure 5b. However, overcutting occurs in the initial position region of the cup-shaped grinding wheel arc-shaped edge. When the roller cuts into the grinding wheel at the Z-axis top of the arc-shaped edge during truing, the swing angle error γ does not affect the profile accuracy of the arc-shaped edge, but it causes the truing region position to change, and overcutting is likely to occur at the beginning and end of the truing trajectory.

### 3.2. The Tool Alignment Error

**Figure 6:**
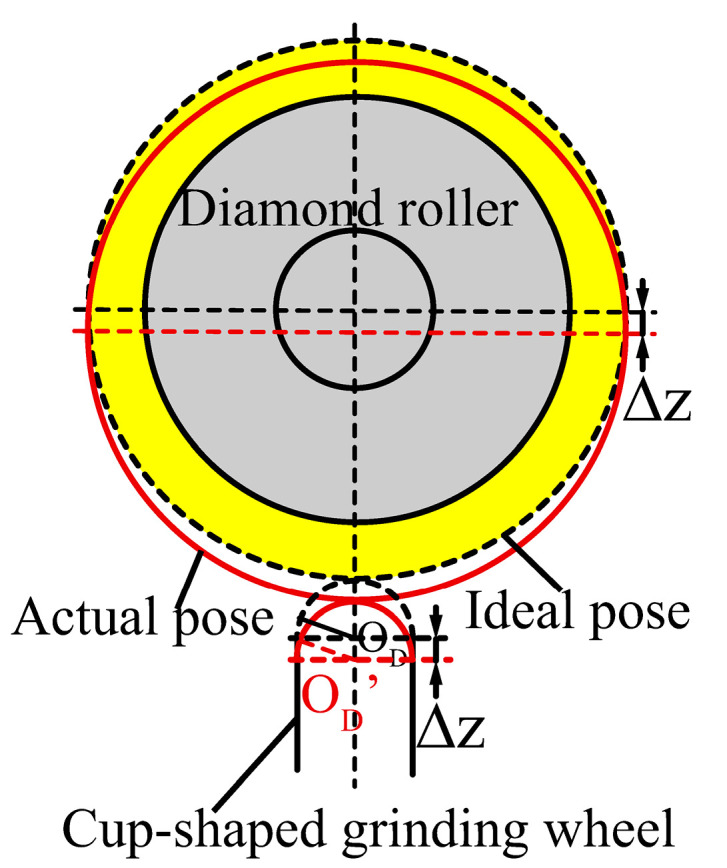
The effect of tool setting errors in the Z direction on the centre position of the arc-shaped cutting edge of cup-shaped grinding wheels.

According to the principle of cup-shaped grinding wheel rolling and truing, the Z-direction tooling setting error only changes the position of the centre of the arc cutting edge of the cup-shaped grinding wheel, without causing profile errors, as shown in Figure 6.

**Figure 7:**
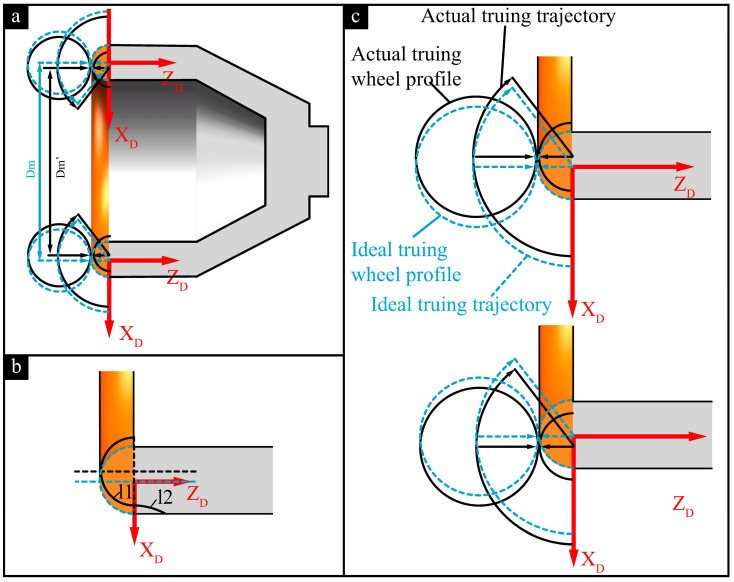
The effect of tool setting errors in the X direction on the truing results. (**a**) Effect of tool setting error in X-axis direction on profile accuracy. (**b**) Composition of the arc-shaped cutting edge of the grinding wheel after truing. (**c**) Effect of tool setting error in X-directions on the mean diameter of grinding wheels.

Figure 7 shows the deviation of the grinding wheel’s arc-shaped cutting edge centre caused by tooling error in the X-direction. This deviation also results in overcutting between the diamond roller and the grinding wheel’s arc-shaped cutting edge during truing. Consequently, the grinding wheel’s profile comprises two arcs of differing radii, l_1_ and l_2_, corresponding, respectively, to the ideal radius and the diamond roller radius as shown in Figure 7b.

In summary, the X-direction tooling setting error does not affect the grinding wheel cutting edge profile in the non-overcutting area, and the impact on the grinding process can be avoided in the overcutting area. However, while the X-direction tool setting error has a limited effect on the cutting edge profile, it significantly impacts the grinding wheel’s mean diameter, as shown in Figure 7a.

**Figure 8:**
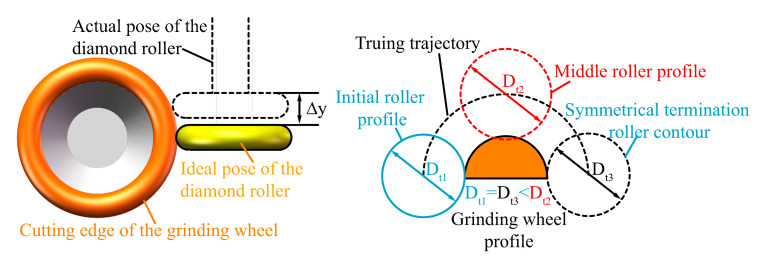
The effect of tool setting errors in the Y direction on the truing results.

The purpose of the tool setting along the Y-axis is to ensure that the distance between the diamond roller’s contact point with the arc-shaped edge of the cup-shaped grinding wheel and the centre of the ideal profile remains consistent throughout the entire motion trajectory. Δy represents the tool setting error in the Y-direction. When Δy is positive, the truing axis motion trajectory is above the horizontal reference plane section; when negative, it is below the horizontal reference plane section. The positive or negative value of the Y-direction error does not affect the influence of this error on the accuracy of the arc-shaped edge profile. When the diamond roller cuts in from the top of the Z-axis, the actual profile of the cup-shaped grinding wheel’s arc-shaped cutting edge is larger than the ideal profile, as shown in Figure 8. The theoretical profile error (PV) value is:

(7) $$\mathrm{PV}=\left|R_{e}-r_{\mathrm{to}}-(R_{e}-\sqrt{{\left(r_{\mathrm{to}}\right)}^{2}-{\left(\Delta y\right)}^{2}})\right|,\;R_{e}=R_{c}-R_{m}$$

where r_to_ is the radius of the arc-shaped cutting edge on a diamond roller, and R_m_ is the mean diameter of the diamond roller. Tooling setting errors cannot be eliminated, but can be reduced to within the required accuracy range through precise detection and compensation truing.

Errors in the Y-direction can directly affect the profile accuracy of the cutting edge after truing. However, selecting an appropriate arc radius r_to_ for the side of the diamond roller can mitigate the impact of Y-direction tool setting errors on profile accuracy. Calculate the derivative of PV with respect to r_to_ to determine the trend of changes, demonstrated by Equation (8).

(8) $$\frac{\mathrm{dPV}}{{\mathrm{dr}}_{\mathrm{to}}}=1-\frac{r_{\mathrm{to}}}{\sqrt{{r_{\mathrm{to}}}^{2}-{\left(\Delta y\right)}^{2}}}$$

where

$$\mathrm{PV}=r_{\mathrm{to}}-\sqrt{{r_{\mathrm{to}}}^{2}-{\left(\Delta y\right)}^{2}}$$

When Δy ≠ 0, r_to_ > Δy

$$r_{\mathrm{to}}>\sqrt{{r_{\mathrm{to}}}^{2}-{\left(\Delta y\right)}^{2}},\;\frac{r_{\mathrm{to}}}{\sqrt{{r_{\mathrm{to}}}^{2}-{\left(\Delta y\right)}^{2}}}>1$$

We can deduce that:

$$\frac{\mathrm{dPV}}{{\mathrm{dr}}_{\mathrm{to}}}<0$$

Based on the above derivation process, when r_to_ > Δy and Δy ≠ 0, it can be seen that as r_to_ increases, the value of PV decreases. At the same time, to prevent interference between the diamond roller and the grinding wheel during truing, the maximum value of r_to_ must be less than the inner diameter of the cup-shaped grinding wheel.

## 4. Experimental Method

### 4.1. Experimental Setup

**Figure 9:**
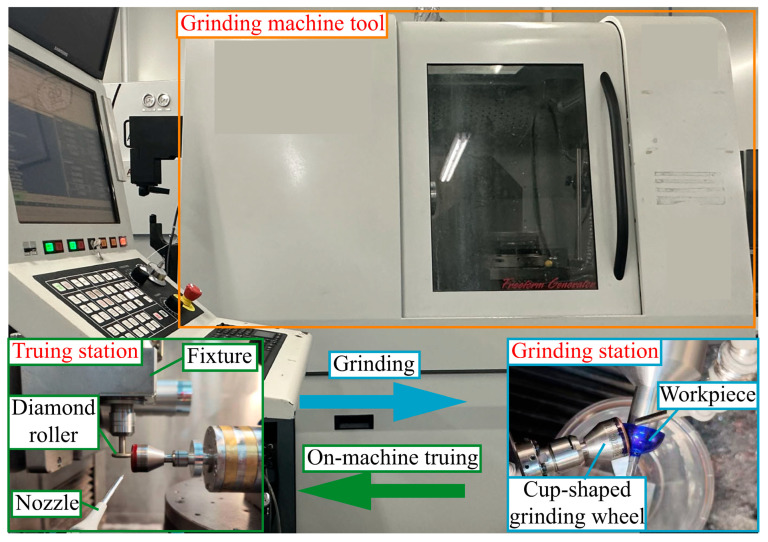
Experimental setup for on-machine truing cup-shaped grinding wheel and grinding complex surfaces.

All experiments are performed on a Moore Machine Tool 350 FG (Bridgeport, CT, USA). The actual mounting positions of the grinding wheel spindle and truing spindle are shown in Figure 9. The axis of the high-speed air-bearing spindle driving the grinding wheel runs parallel to the Z-axis. The truing spindle is mounted on the machine tool via a fixture, with its axis adjusted to run parallel to the Y-axis and capable of vertical movement along this direction. A resin-bond and 5 μm diamond abrasive grains with a 100% concentration were used during the cup-shaped wheel shaping process to achieve high surface quality and shape accuracy. Detailed information on the truing conditions and parameters is provided in Table 1 and Table 2.

The truing accuracy and radius of the cup-shaped grinding wheel’s arc-shaped edge are evaluated using a Talysurf (Berwyn, PA, USA) PGI1240 contact-type profile measuring instrument. A complex structural component is used as the workpiece in the grinding experiment to investigate the grinding performance of the cup-shaped grinding wheel’s arc-shaped edge.

### 4.2. Experimental Procedure

The impact of errors on the truing accuracy of the arc-shaped cutting edge of cup-shaped grinding wheels can be divided into profile errors and dimensional errors. The dimensional errors can be subdivided into cutting-edge dimensional errors and cup-shaped grinding wheel dimensional errors. Among these, the X-direction tool setting error primarily causes deviation in the dimensions D_m_ of the cup-shaped grinding wheel and does not affect profile accuracy during grinding. Therefore, the error is not discussed in this paper. The dimensional error of the diamond roller will be equally reflected in the dimensional error of the cup-shaped grinding wheel cutting edge. This error will be eliminated by gradually measuring and compensating for the arc interpolation radius value R_c_.

The tool setting error in the Y-direction causes profile errors in the grinding wheel’s cutting edge. However, according to the mathematical model, Δy is much smaller than the profile value, so the profile error caused by this error can be ignored. However, the pose errors α and β between the grinding wheel axis and the diamond roller axis are important factors affecting the profile error of the cup-shaped grinding wheel arc cutting edge. In the experiment, the influence of pose error β can be eliminated by adjusting the posture of the truing fixture using an inductive micrometre. However, pose error α is the relative positional error between the two rotational axes in space, which is difficult to measure and adjust. Therefore, a precise compensation method has been proposed based on the error analysis results. By comparing the actual profile with the ideal profile, the profile error of the diamond grinding wheel is obtained. Precise compensation of the error is achieved by adjusting the pitch angle of the grinding wheel spindle.

## 5. Results and Discussion

### 5.1. Analysis and Verification of the Truing Accuracy of the Arc-Shaped Cutting Edge of Cup-Shaped Grinding Wheels

**Figure 10:**
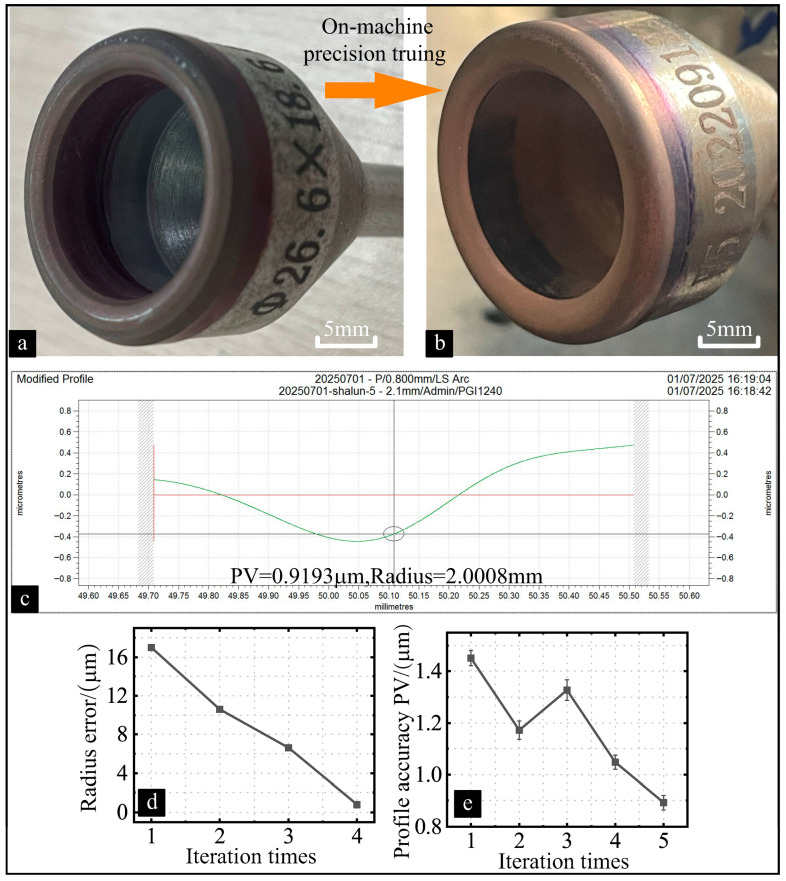
Profile error and radius change during the compensation truing process. Comparing processing quality of the complex structural component before (**a**) and after (**b**) ultra-precision truing. (**c**) The radius and profile error of the most promising grinding wheel. (**d**) Radius error change during the compensation truing process. (**e**) Profile error change during the compensation truing process.

According to the error analysis, the dimensional error of the diamond roller is the primary factor affecting the dimensional accuracy of the arc-shaped edge, and the magnitude of this error is equally reflected in the dimensional error of the cup-shaped grinding wheel cutting edge. This relationship is caused by the fact that the radius Rc of the roller’s circular interpolation motion trajectory is fixed. This value is determined by the combination of the radius of the diamond roller and that of the circular cutting edge. Consequently, as the radius of the diamond roller changes, so does the radius of the grinding wheel’s arc-shaped cutting edge. Through multiple iterations of truing and testing, the average dimensional error of the arc-shaped cutting edge of the cup-shaped grinding wheel was reduced to 1.5 μm, as shown in Figure 10d. Based on experiments compensating for arc edge dimensional error, the established mathematical prediction model indicates that the pose error between the truing spindle and the grinding wheel spindle is the primary factor affecting profile accuracy. Due to the pose error between the grinding wheel spindle and the truing spindle, the cross-section of the diamond roller along its motion trajectory becomes elliptical. The pose error’s angular deviation determines this ellipse’s dimensions and orientation. The actual grinding wheel profile obtained is the inner envelope line of the elliptical trajectory. The non-uniform radii along this trajectory result in profile errors in the wheel with the arc-shaped cutting edge. After initial on-machine precision truing, the profile error of the cup-shaped grinding wheel’s arc-shaped cutting edge is obtained, and the compensation amount for the positional error is determined. Subsequently, the profile error PV was compensated by re-adjusting the pitch angle of the grinding wheel, thereby reducing the average error value from 1.46 μm to 0.89 μm, as shown in Figure 10e.

### 5.2. Grinding of Complex Fused Silica Component

**Figure 11:**
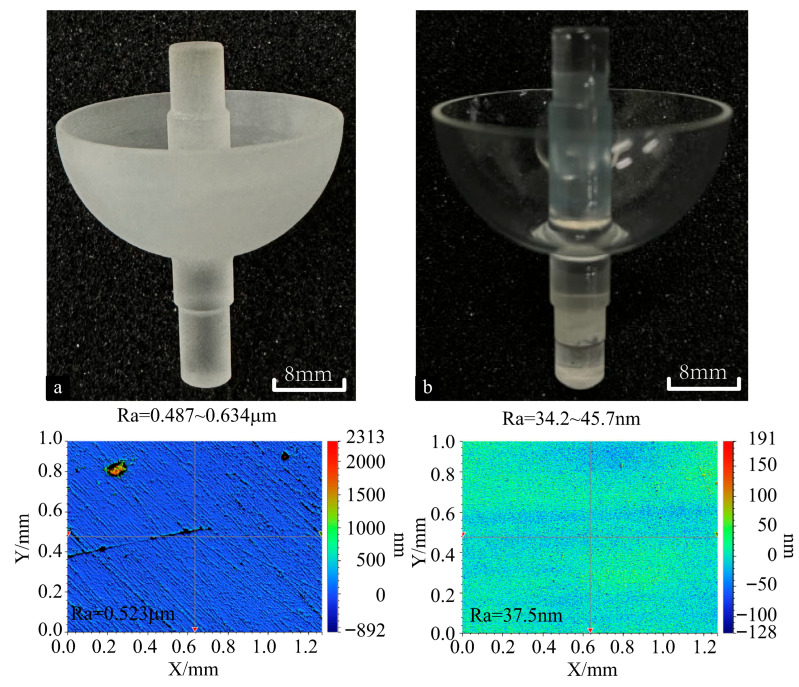
Comparing the processing quality of the complex structural component before (**a**) and after (**b**) ultra-precision grinding with the trued grinding wheel.

To investigate the grinding performance of cup-shaped grinding wheels after truing, grinding experiments are conducted using a workpiece with a complex structure, as shown in Figure 11. The grinding parameters were as follows: a grinding wheel spindle speed of 14,000 rpm, a workpiece spindle speed of 100 rpm, and a grinding depth of 0.1 μm. Figure 11a indicates that the precise mating component with profile error (PV) does not meet the requirements. Additionally, a longer polishing time is required due to its high surface roughness. Figure 11b shows the ultra-precision component processed using the on-machine truing cup-shaped grinding wheel. After ultra-precision grinding, the component’s surface roughness (Ra) is 45.7 nm, respectively. Based on the above results and analysis, the performance of the on-machine truing cup-shaped diamond grinding wheel is acceptable. It should be noted that the surface grinding results for complex components are not uniformly consistent. This is attributable to the varying linear velocities of the workpiece along its axial direction, which leads to differences in the linear velocity ratio between the grinding wheel and the workpiece. Therefore, subsequent research will refine the evaluation system for truing schemes based on the wear rates of diamond and cup-shaped grinding wheels during the truing process. Secondly, it will clarify the impact of different machining parameters on truing accuracy. Finally, it will investigate the effect of linear speed ratios on component surface texture to enhance grinding precision.

## 6. Conclusions

This paper presents an on-machine truing method that is proposed for the precision truing of diamond grinding wheels. The results of the study can be summarised as follows:

(1) Various errors occur during precision truing that significantly impact the profile accuracy of diamond grinding wheels. The primary errors include tooling setting errors, pose errors, and measurement errors in determining the roller radius. Theoretical models of these errors have been developed, and the errors have been categorised and traced to minimise their effect on profile accuracy.
(2) More factors affect cup-shaped grinding wheels’ cutting edge profile accuracy than dimensional accuracy. The main factor affecting profile accuracy is the pose error between the rotating axes of the cup-shaped grinding wheel and the diamond roller. It is worth noting that the larger the dimension of the diamond roller, the greater the impact of pose error on profile accuracy. However, the factor with the greatest impact on dimensional accuracy is the measurement error of the diamond roller dimension.
(3) Compensation truing experiments are conducted based on the error analysis model, resulting in high-precision cup-shaped grinding wheels with arc-shaped cutting edges. Following compensation, the dimensional error of the diamond grinding wheel’s cutting edge decreased by around 92% (from 17.3 μm to 1.5 μm). Profile error is reduced from 1.46 μm to 0.89 μm.

## Figures and Tables

**Table 1:** Truing conditions.

Parameter		
Cup-shaped grinding wheel	Bond	Resin-bond
	Grain size	5 μm
	Diameter D_m_	22.6 mm
	Expected radius	2 mm
Diamond roller	Bond	Metal bond
	Grain size	60 μm
	Diameter D_t_	19 mm
	Radius r_to_	2 mm

**Table 2:** Truing parameters.

Parameter	Value
Speed of the cup-shaped grinding wheel (rpm)	14,000
Speed of the diamond roller (rpm)	500
Truing depth per pass (μm)	2

## Data Availability

Data is contained within the article.
